# A Grey NGM(1,1, *k*) Self-Memory Coupling Prediction Model for Energy Consumption Prediction

**DOI:** 10.1155/2014/301032

**Published:** 2014-06-18

**Authors:** Xiaojun Guo, Sifeng Liu, Lifeng Wu, Lingling Tang

**Affiliations:** ^1^College of Economics and Management, Nanjing University of Aeronautics and Astronautics, Nanjing 211106, China; ^2^School of Science, Nantong University, Nantong 226019, China; ^3^School of Electrical and Computer Engineering, Cornell University, Ithaca, NY 14853, USA

## Abstract

Energy consumption prediction is an important issue for governments, energy sector investors, and other related corporations. Although there are several prediction techniques, selection of the most appropriate technique is of vital importance. As for the approximate nonhomogeneous exponential data sequence often emerging in the energy system, a novel grey NGM(1,1, *k*) self-memory coupling prediction model is put forward in order to promote the predictive performance. It achieves organic integration of the self-memory principle of dynamic system and grey NGM(1,1, *k*) model. The traditional grey model's weakness as being sensitive to initial value can be overcome by the self-memory principle. In this study, total energy, coal, and electricity consumption of China is adopted for demonstration by using the proposed coupling prediction technique. The results show the superiority of NGM(1,1, *k*) self-memory coupling prediction model when compared with the results from the literature. Its excellent prediction performance lies in that the proposed coupling model can take full advantage of the systematic multitime historical data and catch the stochastic fluctuation tendency. This work also makes a significant contribution to the enrichment of grey prediction theory and the extension of its application span.

## 1. Introduction

As one of the most significant national strategic resources, the energy issue is an important factor which restricts the state economy and social development. Along with the ongoing economic growth and the acceleration of industrialization, energy consumption and production will increase even more rapidly. Therefore, it is meaningful to identify and analyze the energy issue legitimately, especially for predicting the future energy consumption correctly and scientifically. Energy consumption is featured by its information uncertainty and few useful analyzing samples. There exist many influential factors (economy condition, industry framework, climatic variation, government policy and so on), which are difficult to determine how exactly they affect energy consumption [1, 2]. Owing to the apparent uncertain characters embodied in the complicated energy system, the energy consumption prediction can be regarded as a grey system exactly.

Most traditional prediction techniques for time series concentrate mainly on the statistical analysis methods, such as simple regression, multivariate regression, exponential smoothing, and so forth, and possess the advantage of accurately approximating the evolutionary trend [3–5]. Nevertheless, the methods must be accomplished with the assumption of realizing the system structure and the limitation of requiring a large amount of historical data. And owing to the increasing complexity, uncertainty, and chaos of the system's structure, it is very difficult to accurately predict random fields using the traditional statistical methods. To overcome this drawback, the grey systems theory was initially proposed by Deng to study the uncertainty of systems [6]. As an important theoretical component, the grey prediction approach represented by GM(1,1) model can weaken the randomness of original statistical data by means of accumulated generating operation [7]. The superiority of grey models is that they only require a limited amount of statistical data without knowing their statistical distribution. GM(1,1) model has ideal predictive effect for approximate homogenous exponential sequence and has already been effectively utilized in numerous fields, such as social economy, geographical environment, engineering science, public transit, and so on [8–12]. Meanwhile, several improved GM(1,1) prediction models were developed for the prediction of electricity load, energy consumption, and so forth [13–16]. Nevertheless, certain time series of energy system often show large stochastic fluctuations due to some uncertain influence factors and present the characteristics of approximate nonhomogeneous exponential. It is inevitable to generate the apparent modeling errors by using GM(1,1) model, so the grey NGM(1,1, *k*) model appropriate for the approximate nonhomogeneous exponential law with stochastic fluctuation was put forward [17].

On the basis of the retrieved modeling, the self-memory principle of dynamic system was developed firstly by Cao [18]. As a mathematic realization of integrating the deterministic and random theories, the principle is a statistically-dynamic method to solve problems of nonlinear dynamic systems [19, 20]. The self-memory principle can retrieve ideal nonlinear dynamic models by means of the practical observational data. It can overcome not only the weakness as being sensitive to initial value of the initial value problem for differential equations, but also the limitation as irrelevant to mechanism equation due to the utilization of the historical materials. It has been utilized increasingly into time series forecasting in numerous fields from meteorology to engineering to economics [21–23]. In recent years, some scholars preliminarily attempted to introduce the self-memory principle into certain basic grey prediction models. Fan derived the self-memory numerical method for solving GM(1,1) model and established a novel grey model recollecting the last several data [24]. Chen et al. established a coupled equation by combining DHGM(2,2) grey differential equation with the self-memory principle to forecast the flood [25]. Guo et al. established an interval grey number self-memory coupling prediction model based on the grey degree of compound grey number [26]. Accordingly, for the purpose of extending the applicable range of grey prediction model and promoting its predictive performance, the self-memory principle is firstly introduced into grey NGM(1,1, *k*) model.

The purpose of this paper is to construct a novel grey NGM(1,1, *k*) self-memory coupling prediction model appropriate for the approximate nonhomogeneous exponential data sequence with stochastic fluctuation emerging in the energy consumption prediction. The novel prediction model synthesizes the advantages of the self-memory principle and grey NGM(1,1, *k*) model through organically coupling the above two prediction methods. Its excellent predictive performance lies in that the grey model's weakness as being sensitive to initial value can be overcome by using multi-time-point initial field instead of only single-time-point initial field.

The remaining content is organized as follows.  Section 2 provides an overview of the relevant literature on generalized GM(*r*, *h*) model, GM(1,1) model, and NGM(1,1, *k*) model.  Section 3 presents the detailed algorithm of a novel NGM(1,1, *k*) self-memory coupling prediction model and a step-by-step procedure. In Section 4, the illustrative examples of total energy, coal, and electricity consumption prediction in China are adopted to demonstrate the adaptability and effectiveness of the proposed NGM(1,1, *k*) self-memory coupling prediction model. Finally, some conclusions are drawn in Section 5.

## 2. Reviewing the Generalized Grey System Model and *NGM*(1,1, *k*) Model

### 2.1. Generalized Grey System Model and Symbols Description

Assume that the sequence

(1) $$\begin{array}{c} F_{t}^{0}=\left\{f_{t}^{0}\;|\;t\in 1,2,\ldots ,n\right\} \end{array}$$

is an original time series, where *f*_*t*_^0^ denotes the observational data at time *t*; then the first-order accumulated generation (abbreviated as 1-AGO) value *f*_*t*_^1^ of the original time series data *f*_*t*_^0^ is obtained as

(2) $$\begin{array}{c} f_{t}^{1}=\overset{t}{\underset{k=1}{\sum }}f_{k}^{0},\;t=1,2,\ldots ,n. \end{array}$$

And the sequence

(3) $$\begin{array}{c} F_{t}^{1}=\left\{f_{t}^{1}\;|\;t\in 1,2,\ldots ,n\right\} \end{array}$$

is called the 1-AGO time series of original time series *F*_*t*_^0^.

The *r*-order differential equation

(4) drFt1dtr+a1dr−1Ft1dtr−1+⋯+ar−1dFt1dt+arFt1 =b1X11(t)+b2X21(t)+⋯+bh−1Xh−11(t)+bh

is called the whitenization equation of the generalized GM(*r*, *h*) model, where the vectors $-\hat{a}={\left[-a_{1},-a_{2},\ldots ,-a_{r}\right]}^{T}$ and $\hat{b}={\left[b_{1},b_{2},\ldots ,b_{h}\right]}^{T}$ are called developing and driving coefficients vectors, respectively. And there is one dependent variable *F*_*t*_^1^ and *h* − 1 independent variables *X*_1_^1^(*t*), *X*_2_^1^(*t*),…, *X*_*h*−1_^1^(*t*) in (4).

### 2.2. Grey GM(1,1) Model

Particularly when *r* = 1 and *h* = 1 in (4), the first-order differential equation with one dependent variable

(5) $$\begin{array}{c} \frac{dF_{t}^{1}}{dt}+aF_{t}^{1}=b \end{array}$$

is called the whitenization equation of the GM(1,1) model, where the parameter *a* represents the developing coefficient, *b* represents the grey input coefficient, and *F*_*t*_^1^ is the dependent variable with AGO input value *f*_*t*_^1^. Meanwhile the equation

(6) $$\begin{array}{c} f_{t}^{0}+0.5a\left(f_{t}^{0}+f_{t-1}^{0}\right)=b,\;t=1,2,\ldots ,n \end{array}$$

is called the basic form of the GM(1,1) model.

Let sampling time Δ*t* = 1; then by applying the least square method with input data *f*_*t*_^0^ and *f*_*t*_^1^, the parameters *a* and *b* in matrix $\hat{R}$ can be obtained as

(7) $$\begin{array}{c} \hat{R}=\left(\begin{array}{c} \hat{a}\\ \hat{b} \end{array}\right)={\left(B_{1}^{T}B_{1}\right)}^{-1}B_{1}^{T}F_{n}^{0}, \end{array}$$

where

(8) $$\begin{array}{c} F_{n}^{0}=\left[\begin{array}{c} f_{2}^{0}\\ f_{3}^{0}\\ \vdots \\ f_{n}^{0} \end{array}\right],\;\;B_{1}=\left[\begin{array}{cc} -0.5\left(f_{2}^{0}+f_{1}^{0}\right) & 1\\ -0.5\left(f_{3}^{0}+f_{2}^{0}\right) & 1\\ \vdots & \vdots \\ -0.5\left(f_{n}^{0}+f_{n-1}^{0}\right) & 1 \end{array}\right]. \end{array}$$

By making the initial value ${\hat{f}}_{1}^{1}=f_{1}^{0}$, the time response sequence of GM(1,1) model is given by

(9) $$\begin{array}{c} {\hat{f}}_{t+1}^{1}=\left(f_{1}^{0}-\frac{b}{a}\right)e^{-at}+\frac{b}{a},\;\forall t\geq 1, \end{array}$$

and the simulated value ${\hat{f}}_{t+1}^{1}$ of dependent variable *F*_*t*_^1^ can be obtained from (9) accordingly. Consider the inverse accumulated generation operation (abbreviated as IAGO)

(10) $$\begin{array}{c} {\hat{f}}_{t+1}^{0}={\hat{f}}_{t+1}^{1}-{\hat{f}}_{t}^{1},\;\forall t\geq 1; \end{array}$$

then the simulated value ${\hat{f}}_{t+1}^{0}$ of IAGO variable *F*_*t*_^0^ can be obtained.

As mentioned above, GM(1,1) model can be considered as the most simple and special case of the generalized GM(*r*, *h*) model. Conversely, generalized GM(*r*, *h*) model can be treated as the extension of GM(1,1) model. As the basic and core part of grey systems theory, GM(1,1) model is one of the most frequently used grey prediction models for time series. It is based on the grey exponential law resulting from accumulated generating and moving averaging operation, which has an ideal predictive effect for the time series with the approximate homogeneous exponential characteristics. Meanwhile, there exist numerous literatures which play an important role in promoting the predictive performance of GM(1,1) model by means of various kinds of optimization techniques [8, 27, 28].

### 2.3. Grey *NGM*(1,1, *k*) Model

When the traditional GM(1,1) model is used for modeling analysis, it is assumed that the original data sequence must obey approximate homogeneous exponential growth law. However, in fact, the approximate homogeneous exponential data sequence is very limited. As for the time series with the characteristics of approximate nonhomogeneous exponential with stochastic fluctuation, Cui et al. first put forward a novel grey prediction model termed NGM(1,1, *k*) model [17, 29]. Aiming at some defects of parameter setting in original NGM(1,1, *k*) model, Cui and Lu [30] constructed a modified NGM(1,1, *k*) model by optimizing parameters of whitenization differential equation. And then Chen and Wei [31] further optimized the grey derivative of approximate nonhomogeneous index sequence GM(1,1) model, thus perfecting the model parameters.

The first-order differential equation

(11) $$\begin{array}{c} \frac{dF_{t}^{1}}{dt}+aF_{t}^{1}=\gamma t+b \end{array}$$

is called the whitenization equation of the NGM(1,1, *k*) model, where the parameter *γ* represents the control coefficient and *a*, *b* are the same as mentioned above. Meanwhile the equation

(12) $$\begin{array}{c} f_{t}^{0}+0.5a\left(f_{t}^{0}+f_{t-1}^{0}\right)=\gamma t+b,\;t=1,2,\ldots ,n \end{array}$$

is called the basic form of the NGM(1,1, *k*) model.

Let sampling time Δ*t* = 1; then the least square estimate of the parameters sequence *a*, *γ*, *b* is given by

(13) $$\begin{array}{c} \hat{R}=\left(\begin{array}{c} \hat{a}\\ \hat{\gamma }\\ \hat{b} \end{array}\right)={\left(B_{2}^{T}B_{2}\right)}^{-1}B_{2}^{T}F_{n}^{0}, \end{array}$$

where

(14) $$\begin{array}{c} F_{n}^{0}=\left[\begin{array}{c} f_{2}^{0}\\ f_{3}^{0}\\ \vdots \\ f_{n}^{0} \end{array}\right],\;\;B_{2}=\left[\begin{array}{ccc} -0.5\left(f_{2}^{0}+f_{1}^{0}\right) & 2 & 1\\ -0.5\left(f_{3}^{0}+f_{2}^{0}\right) & 3 & 1\\ \vdots & \vdots & \vdots \\ -0.5\left(f_{n}^{0}+f_{n-1}^{0}\right) & n & 1 \end{array}\right]. \end{array}$$

By making the initial value ${\hat{f}}_{1}^{1}=f_{1}^{0}$, the time response sequence of NGM(1,1, *k*) model is given by

(15) f^t+11=(f10−γa+γa2−ba)e−at+γa(t+1) −γa2+ba, ∀t≥1,

and the simulated value ${\hat{f}}_{t+1}^{1}$ of dependent variable *F*_*t*_^1^ can be obtained from (15) accordingly. As mentioned above, the simulated value ${\hat{f}}_{t+1}^{0}$ of IAGO variable *F*_*t*_^0^ can be calculated by IAGO.

NGM(1,1, *k*) model is a sort of significant nonlinear grey prediction model in grey systems theory. It can reflect well the approximate nonhomogeneous exponential characteristic of real time series data, which possesses higher accuracy of simulation and prediction. And the NGM(1,1, *k*) model is still further superior to  GM(1,1) model with respect to applied range and predictive performance for the approximate nonhomogeneous exponential law time series with stochastic fluctuation.

## 3. Novel *NGM*(1,1, *k*) Self-Memory Coupling Prediction Model

### 3.1. Fundamental Principles of Self-Memory Principle of Dynamic System

By introducing the memory concept into physics, the self-memory principle of dynamic system is proposed on the basis that natural and social phenomena are all irreversible. The history information should be investigated fully if we want to realize present system and predict its future. Accordingly, the principle emphasizes the relationship between before and after of system status itself, particularly on the systematic evolution law per se. After the memory function which contains historical information is introduced into the system's dynamic differential equation, it can be transformed into an appropriate difference-integral equation which is called a self-memorization one by defining the inner product in Hilbert space. Because the systematic self-memorization equation contains multiple time-point initial fields instead of only single-time-point initial field, the weakness as being sensitive to initial value of the original dynamic differential equation can be overcome. Then through studying systematic inner memorability, the systematic evolutionary trend can be modeled and predicted. The superiority of utilizing self-memory principle lies in that the systematic predictive ability can be improved by means of not only combining dynamics calculations and estimating parameters of historical data, but also extracting systematic information from historical data in statistics.

Based on the above-mentioned literature analysis, the superior self-memory technique is introduced in this section to support the NGM(1,1, *k*) model so as to devise a novel NGM(1,1, *k*) self-memory coupling prediction model. Let the original time series and the 1-AGO time series be *F*_*t*_^0^ = {*f*_*t*_^0^∣*t* ∈ 1,2,…, *n*} and *F*_*t*_^1^ = {*f*_*t*_^1^∣*t* ∈ 1,2,…, *n*}, respectively. Let *dF*_*t*_^1^/*dt* in the whitenization equation of the NGM(1,1, *k*) model be *F*(*x*, *λ*, *t*); then

(16) $$\begin{array}{c} F\left(x,\lambda ,t\right)=-aF_{t}^{1}+\gamma t+b. \end{array}$$

The differential equation *dF*_*t*_^1^/*dt*, which has been determined by (16), is considered to be the system self-memory dynamic equation of the NGM(1,1, *k*) self-memory coupling prediction model:

(17) $$\begin{array}{c} \frac{\text{d}x}{\text{d}t}=F\left(x,\lambda ,t\right), \end{array}$$

where *x* is a variable, *λ* is a parameter, *t* is the time interval series, and *F*(*x*, *λ*, *t*) is the dynamic kernel. Introduce a memory function *β*(*t*) and define an inner product in the Hilbert space:

(18) $$\begin{array}{c} \left(f,g\right)=\overset{b_{0}}{\underset{a_{0}}{\int }}f\left(\xi \right)g\left(\xi \right)\text{d}\xi \;\left(f,g\in L^{2}\right). \end{array}$$

### 3.2. Coupling Modeling Process of *NGM*(1,1, *k*) Model and Self-Memory Principle

Then, the step-by-step modeling procedure of a novel NGM(1,1, *k*) prediction model coupled with self-memory principle is described as follows.

**Step 1 (deducing the difference-integral equation).**

Let one time set *T* = [*t*_−*p*_, *t*_−*p*+1_,…, *t*_−1_, *t*_0_, *t*], where *t*_−*p*_, *t*_−*p*+1_,…, *t*_−1_, *t*_0_ is historical observation time, *t*_0_ is predicted initial time, *t* is coming prediction time, the retrospective order of the equation is *p*, and time sampling interval is Δ*t*.

Apply the above inner product operation into (17) and suppose that variables *x*,  *β* are continuous, differentiable, and integrable; the analytic formula of (17) is therefore obtained as

(19) $$\begin{array}{c} \overset{t}{\underset{t_{-p}}{\int }}\beta \left(\tau \right)\frac{\partial x}{\partial \tau }\text{d}\tau =\overset{t}{\underset{t_{-p}}{\int }}\beta \left(\tau \right)F\left(x,\lambda ,\tau \right)\text{d}\tau ; \end{array}$$

that is,

(20) ∫t−pt−p+1β(τ)∂x∂τdτ+∫t−p+1t−p+2β(τ)∂x∂τdτ+⋯+∫t0tβ(τ)∂x∂τdτ =∫t−ptβ(τ)F(x,λ,τ)dτ.

For every integral term in the left-hand side of (20), after integration by parts, applying the median theorem and performing algebra operation, a difference-integral equation is deduced as

(21) βtxt−β−px−p−∑i=−p0xim(βi+1−βi)  −∫t−ptβ(τ)F(x,λ,τ)dτ=0,

where *β*_*t*_ ≡ *β*(*t*), *x*_*t*_ ≡ *x*(*t*), *β*_*i*_ ≡ *β*(*t*_*i*_), *x*_*i*_ ≡ *x*(*t*_*i*_), *i* = −*p*, −*p* + 1,…, 0, and midvalue *x*_*i*_^*m*^ ≡ *x*(*t*_*m*_), *t*_*i*_ < *t*_*m*_ < *t*_*i*+1_.

**Step 2 (discretizing the self-memory prediction equation).**

Let *x*_−*p*_ ≡ *x*_−*p*−1_^*m*^ and let *β*_−*p*−1_ ≡ 0; then (21) can be converted into

(22) xt=1βt∑i=−p−10xim(βi+1−βi)+1βt∫t−ptβ(τ)F(x,λ,τ)dτ=S1+S2,

which is called the self-memory equation with the retrospective order *p*. As the first term *S*_1_ in (22) denotes the relative contributions of historical data at *p* + 1 times to the value of variable *x*_*t*_, it is defined as the self-memory term. The second term *S*_2_ is the total contribution of the function *F*(*x*, *λ*, *t*) in the retrospective time interval [*t*_−*p*_, *t*_0_], and it is defined as the exogenous effect term. Equation (22) emphasizes serial correlation of the system by itself, that is, the self-memory characteristic of the system. Therefore, it is the self-memory prediction equation of the system.

If integral operation is substituted by summation and differential is transformed into difference in (25), then the midvalue *x*_*i*_^*m*^ is replaced simply by two values of different times; namely,

(23) $$\begin{array}{c} x_{i}^{m}=\frac{1}{2}\left(x_{i+1}+x_{i}\right)\equiv y_{i}. \end{array}$$

By taking equidistance time interval Δ*t*_*i*_ = *t*_*i*+1_ − *t*_*i*_ = 1 and merging *β*_*t*_ and *β*_*i*_ together the self-memory equation of discrete form is shown as follows:

(24) $$\begin{array}{c} x_{t}=\overset{-1}{\underset{i=-p-1}{\sum }}\alpha_{i}y_{i}+\overset{0}{\underset{i=-p}{\sum }}\theta_{i}F\left(x,\lambda ,i\right), \end{array}$$

where *α*_*i*_ = (*β*_*i*+1_ − *β*_*i*_)/*β*_*t*_ and *θ*_*i*_ = *β*_*i*_/*β*_*t*_. *α*_*i*_ and *θ*_*i*_ are called memory coefficients, and *F*(*x*, *λ*, *t*) is determined by the dynamic kernel −*aF*_*t*_^1^ + *bt* + *c* of NGM(1,1, *k*) model.

**Step 3 (solving the self-memory prediction model).**

Assume that there are *L* items of historical data; the memory coefficients *α*_*i*_ and *θ*_*i*_ can be estimated by the least square method. Let

(25) $$\begin{array}{c} \underset{L\times 1}{X_{t}}=\left[\begin{array}{c} x_{t1}\\ x_{t2}\\ \vdots \\ x_{tL} \end{array}\right];\\ \underset{L\times (p+1)}{Y}=\left[\begin{array}{cccc} y_{-p-1,1} & y_{-p,1} & \cdots & y_{-1,1}\\ y_{-p-1,2} & y_{-p,2} & \cdots & y_{-1,2}\\ \vdots & \vdots & \ddots & \vdots \\ y_{-p-1,L} & y_{-p,L} & \cdots & y_{-1,L} \end{array}\right],\\ \underset{(p+1)\times 1}{A}=\left[\begin{array}{c} \alpha_{-p-1}\\ \alpha_{-p}\\ \vdots \\ \alpha_{-1} \end{array}\right],\\ \underset{L\times (p+1)}{\Gamma }=\left[\begin{array}{cccc} F{\left(x,\lambda ,-p\right)}_{1} & F{\left(x,\lambda ,-p+1\right)}_{1} & \cdots & F{\left(x,\lambda ,0\right)}_{1}\\ F{\left(x,\lambda ,-p\right)}_{2} & F{\left(x,\lambda ,-p+1\right)}_{2} & \cdots & F{\left(x,\lambda ,0\right)}_{2}\\ \vdots & \vdots & \ddots & \vdots \\ F{\left(x,\lambda ,-p\right)}_{L} & F{\left(x,\lambda ,-p+1\right)}_{L} & \cdots & F{\left(x,\lambda ,0\right)}_{L} \end{array}\right],\\ \underset{(p+1)\times 1}{\Theta }=\left[\begin{array}{c} \theta_{-p}\\ \theta_{-p+1}\\ \vdots \\ \theta_{0} \end{array}\right], \end{array}$$

then (24) can be expressed as matrix form as follows:

(26) $$\begin{array}{c} X_{t}=YA+\Gamma \Theta . \end{array}$$

Let *Z* = [*Y*, Γ] and $W=\left[\begin{array}{c} A\\ \Theta \end{array}\right]$; then (26) turns into

(27) $$\begin{array}{c} X_{t}=ZW; \end{array}$$

thereby *W* is obtained by the least square method:

(28) $$\begin{array}{c} W={\left(Z^{T}Z\right)}^{-1}Z^{T}X_{t}. \end{array}$$

When the memory coefficient matrix *W* is obtained, the simulating and predicting of original data sequence *F*_*t*_^0^ can be carried out. For the simulated and predicted values ${\hat{f}}_{t}^{1}$ of the first-order accumulated generation sequence in NGM(1,1, *k*) self-memory coupling model, their inverse accumulated values ${\hat{f}}_{t}^{0}$ can be obtained as follows:

(29) $$\begin{array}{c} {\hat{f}}_{t}^{0}={\hat{f}}_{t}^{1}-{\hat{f}}_{t-1}^{1}, \end{array}$$

where *t* = 1,2,…, *n* and ${\hat{f}}_{0}^{1}\equiv 0$.

**Step 4 (modeling simulation and prediction accuracy check).**

The absolute percentage error (abbreviated as APE) at time *t* is denoted by

(30) $$\begin{array}{c} \text{AP}{\text{E}}_{t}=\frac{\left|{\hat{f}}_{t}^{0}-f_{t}^{0}\right|}{f_{t}^{0}}\times 100\%, \end{array}$$

and the mean absolute percentage error (abbreviated as MAPE) at all times is defined as

(31) $$\begin{array}{c} \text{MAPE}=\frac{1}{n}\overset{n}{\underset{t=1}{\sum }}\text{AP}{\text{E}}_{t}. \end{array}$$

Accordingly, the comparison analysis between actual values and simulative values derived from each prediction model can be analyzed using APE_*t*_ and MAPE values.

At the same time, the established NGM(1,1, *k*) self-memory coupling prediction model must pass the simulation accuracy check before performing extrapolation and prediction. We usually check their accuracy by methods such as the “posterior variance ratio” and “small error probability” according to Table 1.

$S_{1}=\sqrt{\left(1/n\right)\sum_{t=1}^{n}{\left(f_{t}^{0}-{\overset{-}{f}}_{t}^{0}\right)}^{2}}$ and $S_{2}=\sqrt{\left(1/n\right)\sum_{t=1}^{n}{\left(\varepsilon (k)-\overset{-}{\varepsilon }\right)}^{2}}$ are the mean square error of original values and residual error, respectively. For a given *C*_0_ > 0, if the posterior variance ratio *C* = *S*_2_/*S*_1_ < *C*_0_, then the model is considered to pass through the posterior variance ratio check [7].

In the same way, for a given *p*_0_ > 0, if the small error probability $p=P(\left|\varepsilon (k)-\overset{-}{\varepsilon }\right|<0.6745S_{1})>p_{0}$, then the model is supposed to pass through the small error probability check [7].

### 3.3. Programming Procedure of Coupling Prediction Model

**Figure 1:**
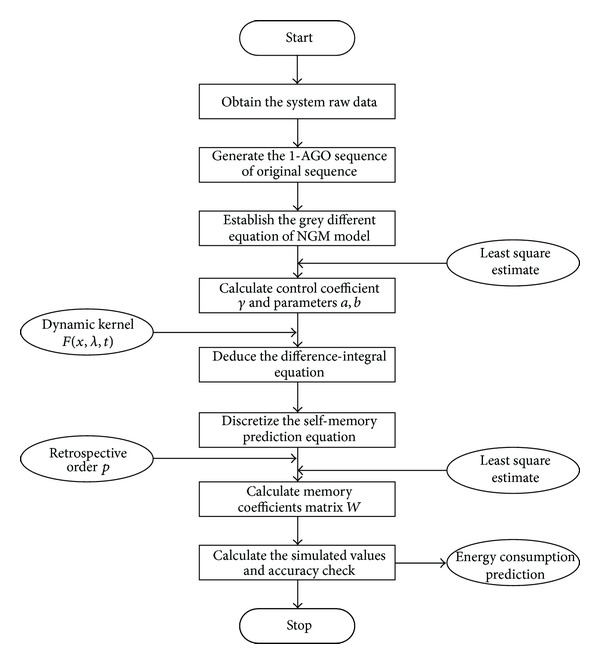
Programming flowchart for NGM(1,1, *k*) self-memory coupling prediction model.

The calculation process is carried out as mentioned above with the help of Matlab software in order to save the computational effort. And the programming flowchart for NGM(1,1, *k*) self-memory coupling prediction model is shown in Figure 1.

## 4. Illustrative Examples for Energy Consumption Prediction

**Figure 2:**
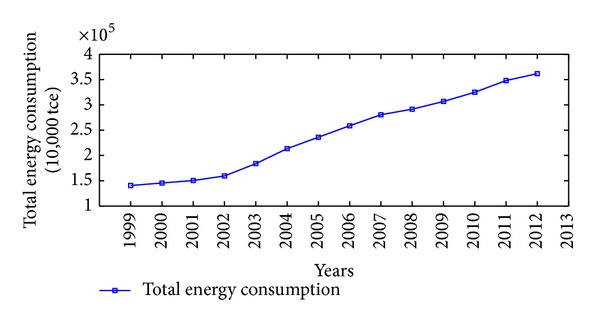
Time series of total energy consumption (10,000 tce) in China from 1999 to 2012.

**Figure 3:**
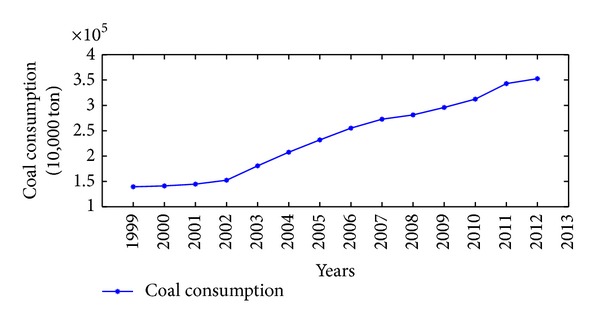
Time series of coal consumption (10,000 ton) in China from 1999 to 2012.

**Figure 4:**
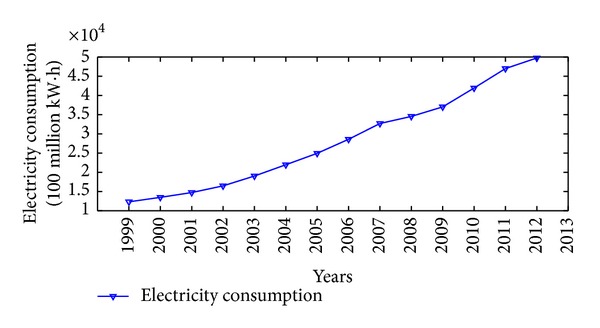
Time series of electricity consumption (100 million kW·h) in China from 1999 to 2012.

China is one of the major countries in energy consumption. According to China's energy consumption structure, coal is the main primary energy and electricity is the main secondhand energy. Therefore, the illustrative examples of total energy consumption, coal consumption, and electricity consumption in China are adopted to verify the effectiveness and practicability of the proposed NGM(1,1, *k*) self-memory coupling prediction model (abbreviated as NGM + self-memory model). According to the China Energy Statistical Yearbook 2013, Figures 2, 3, and 4 show the annual consumption of total energy, coal, and electricity in China from 1999 to 2012, respectively. As it is seen in Figures 2–4, there has been a tremendous rise in energy consumption for each energy source, accompanied by irregular fluctuations due to the unstable changes occurring in the social and economic factors. In conclusion, China's energy consumption shows an obvious nonhomogeneous exponential rising tendency with stochastic fluctuation.

In the statistical models, Markov chain can be used to explain the stochastic fluctuation phenomenon in which state transfer probability matrix is the basis of Markov prediction model. In general, we cannot determine the typical distribution of random variables and can only use the frequency instead of the probability. Probability theory points out that this replacement is meaningful only on the premise of large sampled values. On the contrary, the grey prediction models just possess an apparent superiority over a limited amount of statistical data without knowing their statistical distribution. Thereinto, the NGM(1,1, *k*) model is especially appropriate for the approximate nonhomogeneous exponential law time series with stochastic fluctuation.

Following the coupling modeling process as mentioned above, the NGM(1,1, *k*) self-memory coupling prediction models for total energy, coal, and electricity consumption are established to model and predict the consumption amounts for the upcoming three years, respectively. At the same time, these novel models are compared with the traditional GM(1,1) model (abbreviated as GM model), the GM(1,1) model with three-point moving average (abbreviated as GM + three-point model) [32], and the traditional NGM(1,1, *k*) model (abbreviated as NGM model) to perform the error analysis. APE_*t*_ and MAPE are used to compare the actual values with simulative values to evaluate the predictive performance of novel NGM + self-memory model over other popular grey models.

### 4.1. Total Energy Consumption Forecasting in China

Based on statistical data from 1999 to 2012, the differential equation of NGM model is formulated as follows:

(32) $$\begin{array}{c} \frac{\text{d}F_{t}^{1}}{\text{d}t}=-0.0199F_{t}^{1}+22066.6569t+90668.0270. \end{array}$$

If the right-side terms of (32) are regarded as the dynamic kernel *F*(*x*, *λ*, *t*), then d*F*_*t*_^1^/d*t* = *F*(*x*, *λ*, *t*). The self-memorization equation can be established for total energy consumption forecasting. The value of retrospective order is determined as *p* = 1 by trial calculation method under the principle of minimum error. After the differential equation is dealt with discretely, the memory coefficients can be solved by the least square method. Then the prediction equation of total energy consumption can be expressed as

(33) $$\begin{array}{c} x_{t}=\overset{-1}{\underset{i=-2}{\sum }}\alpha_{i}y_{i}+\overset{0}{\underset{i=-1}{\sum }}\theta_{i}F\left(x,\lambda ,i\right), \end{array}$$

where *α*_−2_ = 0.4493,  *α*_−1_ = 0.4977,  *θ*_−1_ = 4.2646, and *θ*_0_ = −1.6101.

Through calculation, the actual and simulative values of four different models are presented in Table 2, respectively. It is shown that the simulative MAPE of NGM model is lower than GM model and GM + three-point model. Moreover, the self-memory principle significantly further improves the prediction accuracy of NGM model. Consequently, the NGM + self-memory model yields the lowest MAPE compared with the other popular grey models.

**Figure 5:**
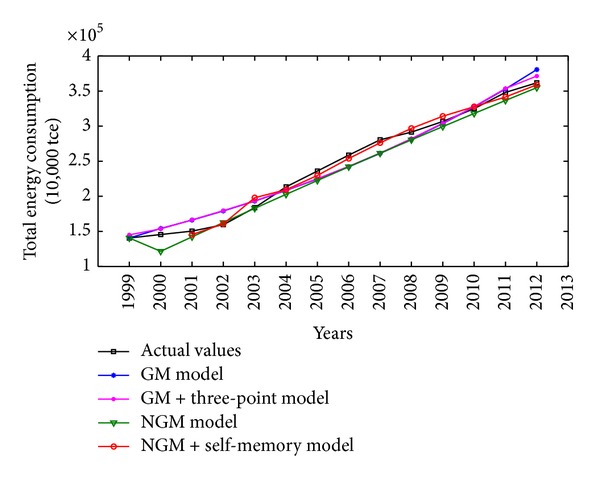
Comparison among original and simulative curves of different models for total energy consumption in China.

The actual values and the simulative results for total energy consumption from 1999 to 2012 obtained by four different grey models are also presented in Figure 5. As can be seen from Table 2 and Figure 5, the NGM + self-memory model can better catch the development tendency of total energy consumption with the characteristics of nonhomogeneous exponential law. And the self-memory principle possesses an apparent advantage over other grey models when dealing with the stochastic fluctuation phenomenon.

### 4.2. Coal Consumption Forecasting in China

Based on statistical data from 1999 to 2012, the differential equation of NGM model is formulated as follows:

(34) $$\begin{array}{c} \frac{\text{d}F_{t}^{1}}{\text{d}t}=-0.0123F_{t}^{1}+21890.3477t+86775.2490. \end{array}$$

If the right-side terms of (34) are regarded as the dynamic kernel *F*(*x*, *λ*, *t*), then d*F*_*t*_^1^/d*t* = *F*(*x*, *λ*, *t*). The self-memorization equation can be established for coal consumption forecasting. The value of retrospective order is determined as *p* = 2 by trial calculation method under the principle of minimum error. After the differential equation is dealt with discretely, the memory coefficients can be solved by the least square method. Then the prediction equation of coal consumption can be expressed as

(35) $$\begin{array}{c} x_{t}=\overset{-1}{\underset{i=-3}{\sum }}\alpha_{i}y_{i}+\overset{0}{\underset{i=-2}{\sum }}\theta_{i}F\left(x,\lambda ,i\right), \end{array}$$

where *α*_−3_ = 0.0717, *α*_−2_ = −0.4124, *α*_−1_ = 1.2960, *θ*_−2_ = −49.6300, *θ*_−1_ = 102.7246, and *θ*_0_ = −51.2947.

Through calculation, the actual and simulative values of four different models are presented in Table 3, respectively. It is shown that the simulative MAPE of NGM model is lower than GM model and GM + three-point model. Moreover, the self-memory principle significantly further improves the prediction accuracy of NGM model. Consequently, the NGM + self-memory model yields the lowest MAPE compared with the other popular grey models.

**Figure 6:**
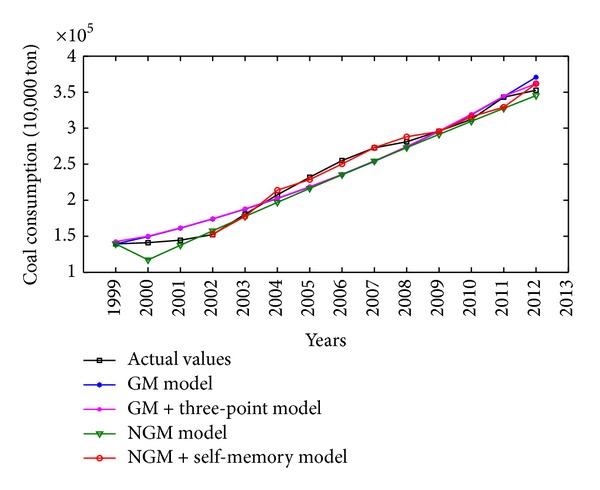
Comparison among original and simulative curves of different models for coal consumption in China.

The actual values and the simulative results for coal consumption from 1999 to 2012 obtained by four different grey models are also presented in Figure 6. As can be seen from Table 3 and Figure 6, the NGM + self-memory model can better catch the development tendency of coal consumption with the characteristics of nonhomogeneous exponential law. And the self-memory principle possesses an apparent advantage over other grey models when dealing with the stochastic fluctuation phenomenon.

### 4.3. Electricity Consumption Forecasting in China

Based on statistical data from 1999 to 2012, the differential equation of NGM model is formulated as follows:

(36) $$\begin{array}{c} \frac{\text{d}F_{t}^{1}}{\text{d}t}=0.0599F_{t}^{1}+1390.9613t+8703.5942. \end{array}$$

If the right-side terms of (36) are regarded as the dynamic kernel *F*(*x*, *λ*, *t*), then d*F*_*t*_^1^/d*t* = *F*(*x*, *λ*, *t*). The self-memorization equation can be established for electricity consumption forecasting. The value of retrospective order is determined as *p* = 2 by trial calculation method under the principle of minimum error. After the differential equation is dealt with discretely, the memory coefficients can be solved by the least square method. Then the prediction equation of electricity consumption can be expressed as

(37) $$\begin{array}{c} x_{t}=\overset{-1}{\underset{i=-3}{\sum }}\alpha_{i}y_{i}+\overset{0}{\underset{i=-2}{\sum }}\theta_{i}F\left(x,\lambda ,i\right), \end{array}$$

where *α*_−3_ = 0.2790, *α*_−2_ = −0.5414, *α*_−1_ = 1.1746, *θ*_−2_ = 14.9812, *θ*_−1_ = −21.8940, and *θ*_0_ = 9.6612.

Through calculation, the actual and simulative values of four different models are presented in Table 4, respectively. It is shown that the simulative MAPE of NGM model is lower than GM model and GM + three-point model. Moreover, the self-memory principle significantly further improves the prediction accuracy of NGM model. Consequently, the NGM model + self-memory model yield the lowest MAPE compared with the other popular grey models.

**Figure 7:**
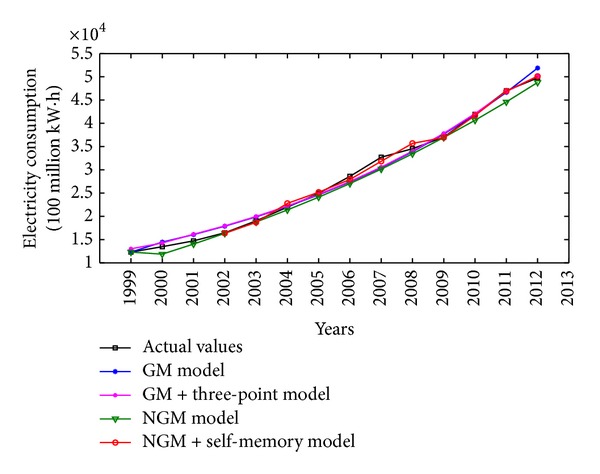
Comparison among original and simulative curves of different models for electricity consumption in China.

The actual values and the simulative results for electricity consumption from 1999 to 2012 obtained by four different grey models are also presented in Figure 7. As can be seen from Table 4 and Figure 7, the NGM + self-memory model can better catch the development tendency of electricity consumption with the characteristics of nonhomogeneous exponential law. And the self-memory principle possesses an apparent advantage over other grey models when dealing with the stochastic fluctuation phenomenon.

The MAPE of different prediction models for total energy, coal, and electricity consumption are all presented in Table 5, respectively. It is obvious that the prediction error of NGM(1,1, *k*) model is lower than GM(1,1) model and GM(1,1) model with three-point, and the self-memory principle further reduces the prediction error of NGM(1,1, *k*) model remarkably.

The “posterior error” method is used to perform the simulation accuracy check, with the results showing that the posterior variance ratio and small error probability of three NGM + self-memory prediction models are all up to the first precision level on the basis of Table 1. The simulation accuracies of the simulative values to the actual values are 97.70%, 98.27%, and 98.46%, respectively. Because three NGM + self-memory prediction models have all passed through the simulation accuracy check, they all could be used to carry out extrapolation and prediction, which could reasonably reflect the growth trend of the future energy consumption of China.

The total energy, coal, and electricity consumption in China from 2013 to 2015 is predicted according to the NGM + self-memory prediction models as mentioned above. The results show that, compared with the data of 2012, the total energy, coal energy, and electricity energy consumption will increase at the annual average rate of 3.44%, 2.53%, and 8.08%, respectively, in the next three years. In the energy consumption structure, the proportion of coal energy and electricity energy consumption will increase gradually, as shown in Table 6. This means that along with the rapid development of China's economy, the need for energy is increasing continuously. Moreover, with the increasing growth of coal energy consumption, the proportion of electricity energy consumption is also growing rapidly with a growth rate far higher than coal energy.

## 5. Conclusion

In this study, aiming at the approximate nonhomogeneous exponential data sequence with stochastic fluctuation emerging in the energy consumption, the predictive performance of the traditional NGM(1,1, *k*) model has been markedly improved by using the self-memory principle of dynamic system. The illustrative examples show the superiority of NGM(1,1, *k*) self-memory coupling prediction model over other popular grey models. This superiority results from the organic integration of the grey NGM(1,1, *k*) model and the self-memory principle. The coupling prediction model can take full advantage of the systematic multitime historical data and tightly catch the stochastic fluctuation tendency. The future total energy, coal, and electricity consumption of China has been effectively predicted using the proposed NGM(1,1, *k*) self-memory coupling prediction model. It is worth popularizing and applying in other relevant energy consumption predictions.

These results may guide China's institutions related to energy production in implementing the energy planning studies and framing the suitable energy strategies. In the future, China's energy consumption structure will still mainly depend on coal energy, together with an obvious upward trend of the consumption share of electricity energy. This conforms to the future sustainable multiple clean energy consumption strategy that is based mainly on coal. Therefore, the proposed coupling prediction model could provide a reference for other countries (especially developing countries) to establish and adjust the energy consumption structure and coordinate the relationship among energy, economy, and environment.

This study also supports that there is still room for improving the performance of the existing prediction methods for energy consumption. As a future work, grey prediction models based on various kinds of optimization techniques will be integrated with the self-memory principle in order to further improve the prediction accuracy and stability in energy consumption. Meanwhile, we have not found an ideal algorithm for the optimal retrospective order, only by means of the trial calculation method under the principle of minimum error. Therefore, whether there are certain intelligent optimization algorithms, such as nonlinear programming and particle swarm optimization, that could be introduced into the coupling model needs further exploration.

## Figures and Tables

**Table 1:** Test list of posterior variance ratio and small error probability.

Modeling accuracy class	Test index	
	Posterior variance ratio *C*	Small error probability *p*
1st level (superior)	≤0.35	≥0.95
2nd level (qualified)	0.35~0.50	0.80~0.95
3rd level (marginal)	0.50~0.65	0.70~0.80
4th level (disqualified)	≥0.65	≤0.70

**Table 2:** Comparison of four different models for total energy consumption in China (unit: 10,000 tce).

Years	Actual values	GM model		GM + three-point model		NGM model		NGM + self-memory model	
		Simulative values	APE_*t*_ (%)	Simulative values	APE_*t*_ (%)	Simulative values	APE_*t*_ (%)	Simulative values	APE_*t*_ (%)
1999	140568.82	140568.82	0.00	145078.39	3.21	140568.82	0.00	—	—
2000	145530.86	154097.54	5.89	153607.37	5.55	121587.39	16.45	—	—
2001	150405.80	166155.76	10.47	166470.28	10.68	142213.09	5.45	145441.98	3.30
2002	159430.99	179157.54	12.37	179496.67	12.59	162614.72	2.00	160778.73	0.85
2003	183791.82	193176.71	5.11	193542.38	5.31	182794.71	0.54	197959.80	7.71
2004	213455.99	208292.90	2.42	208687.18	2.23	202755.47	5.01	209398.51	1.90
2005	235996.65	224591.93	4.83	225017.06	4.65	222499.39	5.72	229729.69	2.66
2006	258676.30	242166.37	6.38	242624.77	6.21	242028.81	6.44	253872.41	1.86
2007	280507.94	261116.02	6.91	261610.29	6.74	261346.08	6.83	276202.44	1.53
2008	291448.29	281548.49	3.40	282081.44	3.21	280453.49	3.77	296765.50	1.82
2009	306647.15	303579.81	1.00	304154.47	0.81	299353.33	2.38	314095.98	2.43
2010	324939.15	327335.10	0.74	327954.72	0.93	318047.84	2.12	327720.81	0.86
2011	348001.66	352949.25	1.42	353617.35	1.61	336539.26	3.29	341540.94	1.86
2012	361732.01	380567.72	5.21	371361.56	2.66	354829.80	1.91	358909.33	0.78
MAPE (%)			4.95		4.74		4.42		2.30

There are no simulative values from year 1999 to 2000 owing to the retrospective order *p* = 1.

**Table 3:** Comparison of four different models for coal consumption in China (unit: 10,000 ton).

Years	Actual values	GM model		GM + three-point model		NGM model		NGM + self-memory model	
		Simulative values	APE_*t*_ (%)	Simulative values	APE_*t*_ (%)	Simulative values	APE_*t*_ (%)	Simulative values	APE_*t*_ (%)
1999	139336.46	139336.46	0.00	142733.77	2.44	139336.46	0.00	—	—
2000	141091.70	149528.38	5.98	150048.17	6.35	117190.05	16.94	—	—
2001	144528.11	161279.66	11.59	161587.50	11.80	137509.12	4.86	—	—
2002	152282.66	173954.46	14.23	174286.49	14.45	157579.06	3.48	152937.13	0.43
2003	180587.04	187625.36	3.90	187983.48	4.10	177402.94	1.76	177480.28	1.72
2004	207561.29	202370.64	2.50	202756.91	2.31	196983.77	5/10	213909.03	3.06
2005	231851.07	218274.73	5.86	218691.36	5.68	216324.53	6.70	228641.92	1.38
2006	255065.45	235428.71	7.70	235878.09	7.52	235428.16	7.70	250270.35	1.88
2007	272745.88	253930.81	6.90	254415.50	6.72	254297.57	6.76	272886.66	0.05
2008	281095.92	273886.97	2.56	274409.74	2.38	272935.63	2.90	288069.05	2.48
2009	295833.08	295411.46	0.14	295975.32	0.05	291345.17	1.52	295298.07	0.18
2010	312236.50	318627.53	2.05	319235.71	2.24	309529.01	0.87	315925.57	1.18
2011	342950.24	343668.14	0.21	344324.11	0.40	327489.91	4.51	329093.20	4.04
2012	352647.07	370676.65	5.11	361673.81	2.56	345230.59	2.10	361701.32	2.57
MAPE (%)			5.13		4.93		4.66		1.73

There are no simulative values from year 1999 to 2001 owing to the retrospective order *p* = 2.

**Table 4:** Comparison of four different models for electricity consumption in China (unit: 100 million  kW*·*h).

Years	Actual values	GM model		GM + three-point model		NGM model		NGM + self-memory model	
		Simulative values	APE_*t*_ (%)	Simulative values	APE_*t*_ (%)	Simulative values	APE_*t*_ (%)	Simulative values	APE_*t*_ (%)
1999	12304.71	12304.71	0.00	13019.57	5.81	12304.71	0.00	—	—
2000	13472.38	14449.29	7.25	14275.93	5.96	11872.22	11.88	—	—
2001	14723.46	16073.79	9.17	16134.67	9.58	14038.59	4.65	—	—
2002	16465.45	17880.94	8.60	17948.66	9.01	16338.71	0.77	16389.34	0.46
2003	19031.60	19891.26	4.52	19966.59	4.91	18780.82	1.32	18696.47	1.76
2004	21971.37	22127.59	0.71	22211.40	1.09	21373.68	2.72	22793.26	3.74
2005	24940.32	24615.35	1.31	24708.58	0.93	24126.62	3.26	25177.32	0.95
2006	28587.97	27382.80	4.22	27486.52	3.85	27049.50	5.38	27923.64	2.32
2007	32711.81	30461.39	6.88	30576.77	6.53	30152.82	7.82	31833.53	2.68
2008	34541.35	33886.11	1.90	34014.45	1.53	33447.72	3.17	35697.92	3.35
2009	37032.14	37695.85	1.79	37838.63	2.18	36946.02	0.23	36937.38	0.26
2010	41934.49	41933.92	0.00	42092.75	0.38	40660.28	3.04	41689.98	0.58
2011	47000.88	46648.47	0.75	46825.15	0.37	44603.84	5.10	46905.23	0.20
2012	49762.64	51893.06	4.28	50144.86	0.77	48790.85	1.95	50099.17	0.68
MAPE (%)			3.83		3.78		3.66		1.54

There are no simulative values from year 1999 to 2001 owing to the retrospective order *p* = 2.

**Table 5:** MAPE of four different models for energy consumption.

Energy sources	GM model	GM + three-point model	NGM model	NGM + self-memory model
Total energy	4.95%	4.74%	4.42%	2.30%
Coal	5.13%	4.93%	4.66%	1.73%
Electricity	3.83%	3.78%	3.66%	1.54%

**Table 6:** The predictive values of the NGM + self-memory model and the growth rates.

Years	Total Energy (10,000 tce)		Coal (10,000 ton)		Electricity (100 million kW*·*h)	
	Quantity	Growth rate	Quantity	Growth rate	Quantity	Growth rate
2013	373905.27	3.37%	357900.57	1.49%	52545.77	5.59%
2014	385239.04	3.03%	364615.31	1.88%	57218.44	8.89%
2015	400317.60	3.91%	380018.09	4.22%	62808.14	9.77%
